# Updated Insights into Probiotics and Hepatobiliary Diseases

**DOI:** 10.3390/biomedicines12030515

**Published:** 2024-02-25

**Authors:** Xiaoyu Xu, Cheng Zhang, Guoyi Tang, Ning Wang, Yibin Feng

**Affiliations:** School of Chinese Medicine, Li Ka Shing Faculty of Medicine, The University of Hong Kong, Hong Kong 999077, China; u3008268@connect.hku.hk (X.X.); zttc@connect.hku.hk (C.Z.); tanggy@connect.hku.hk (G.T.); ckwang@hku.hk (N.W.)

**Keywords:** probiotics, gut microbiota, gut–liver axis, hepatobiliary diseases

## Abstract

Hepatobiliary diseases have a high prevalence worldwide, with a wide range of diseases involved in the liver and biliary system. Modifications in gut microbiota have been proven to have an association with unbalanced intestinal homeostasis and the dysfunction of host metabolism and the immune system, which can be the risk factors for many hepatobiliary diseases, such as nonalcoholic fatty liver disease (NAFLD), alcoholic liver disease (ALD), nonalcoholic fatty steatohepatitis (NASH), hepatitis, cirrhosis, hepatocellular carcinoma (HCC) and cholestasis, as well as infection due to liver transplantation. Probiotics are commonly used gut microbiota-targeted strategies to treat dysbiosis and intestinal dysfunction, as well as the gut–liver axis, which can enhance the effectiveness of probiotics in the management of liver diseases. Recent studies have explored more potential single or mixed strains of probiotics, and bioinformatics methods can be used to investigate the potential mechanisms of probiotics on liver diseases. In this review, we summarize the preclinical and clinical studies on the role of probiotics in hepatobiliary diseases from 2018 to 2023, revealing the possible mechanism of probiotics in the treatment of hepatobiliary diseases and discussing the limitations of probiotics in treating hepatobiliary diseases. This review provides updated evidence for the development of probiotic products, exploration of new probiotic strains, and support for clinical studies. Further studies should focus on the safety, viability, and stability of probiotics, as well as medication dosage and duration in clinical practice.

## 1. Introduction

Hepatobiliary diseases include a wide range of hepatic and biliary disorders and have a high prevalence worldwide, posing health concerns to the public. Notably, liver disease accounts for over two million deaths annually and is a leading cause of death, with cirrhosis and hepatocellular carcinoma (HCC) as the main risk factors [1]. Due to the high prevalence of obesity and type 2 diabetes (T2DM), nonalcoholic fatty liver disease (NAFLD) has rapidly become a global burgeoning health problem and exhibits trends to affect the young generation, as NAFLD is the most common chronic disease in adolescents and children [2,3]. Hepatic steatosis is the early stage and can further progress into nonalcoholic steatohepatitis (NASH), characterized by hepatocyte injury, inflammatory infiltration, and/or collagen deposition [4]. The advanced stage of NASH can lead to cirrhosis, in which the damaged hepatocytes are replaced by scar tissue, and stellate cells are activated [5]. Cirrhosis is a high-risk factor for developing liver cancer, ultimately causing mortality [6]. Liver transplantation is considered necessary when the complications of liver diseases fail to be controlled properly by treatment, and it has been the life-saving option for patients with chronic end-stage liver diseases. However, some challenges limit the use of liver transplantation, such as the recurrence of diseases, long-term maintenance of liver allograft function, and side effects of antirejection drugs [7]. In addition, cholestasis blocks the transportation of bile acid from the liver to the intestine, impairing the function of bile acid entering the liver intestinal circulation and damaging the biliary epithelium in most cases [8,9].

During the past two decades, mounting preclinical and clinical findings demonstrate that gut microbiota, as a dynamic ecosystem, is involved in sustaining physiological homeostasis, such as the immune system and host metabolism [10]. The dysbiosis of gut microbiota is suggested to induce intestinal inflammation and impair the integrity of the intestinal barrier, facilitating the excess of harmful molecules, like lipopolysaccharide (LPS), to translocate to the liver and other organs through the portal vein [11]. In addition to the microbes, the metabolites derived from the microbiota, like bile acids and trimethylamine, are also responsible for the progression of many chronic diseases [12]. Therefore, gut microbiota-related interventions are widely used as alternative treatments for chronic diseases by maintaining the balance of the gut microbiota and improving the gut barrier function, with few adverse events reported [13,14].

Probiotics, prebiotics, and synbiotics have been widely investigated as microbiome-targeted therapies. Probiotics are defined as living microorganisms that exhibit benefits to host health with adequate consumption. Probiotic organisms are mainly derived from the gut and some fermented foods, like yogurts. *Lactobacillus* spp. and *Bifidobacterium* spp. are the main and common genera developed into commercial products, and more species and strains have been accepted as novel probiotic products [15]. Prebiotic generally refers to a substance promoting the growth of probiotics in the gut and immune homeostasis, while a synbiotic is a combination of probiotics and prebiotics, and it guarantees the survival of probiotics and selectively promotes the growth of health-beneficial bacteria [16,17]. Among these, probiotics are the common strategies to treat dysbiosis and intestinal dysfunction, with high research and commercial values, and they are involved in the regulation of the gut–liver axis, which plays an important role in the maintenance of host health.

The previous data demonstrated that the progression of diseases was correlated with gut microbiota alternation, and the strains of bacteria with significant changes between control healthy individuals and patients were recognized as the effective supplement treatment for liver diseases, which provided theoretical evidence supporting further studies and more clinical practice to confirm the role of probiotics was required. For this reason, we performed a literature review about probiotic and hepatobiliary diseases based on the literature published in 2018–2023 in the PubMed and Web of Science databases to gain updated insights into the role of different probiotic strains in the management and treatment of hepatobiliary diseases. Recent studies have explored more single or mixed strains of health-promoting bacteria as an intervention to manage and prevent liver diseases, which may be developed into probiotic products (Table 1). Additionally, the use of probiotics has been investigated to block or delay the progression of HCC by reducing the risk factors like T2DM and NAFLD. With the development of bioinformatic techniques, some bioinformatics methods have been widely used in the identification of the potential mechanisms of gut microbiota modifications by probiotics on liver diseases. In future studies, the safety, viability, and stability of probiotics should be highlighted to increase their effectiveness in preventing and managing liver diseases, and the medication dosage and duration should be further evaluated and confirmed in more clinical practice.

## 2. Probiotics and Nonalcoholic Fatty Liver Disease

Nonalcoholic fatty liver disease (NAFLD) has a high prevalence worldwide due to diet, lifestyle, and work stress in the population of adults; meanwhile, the occurrence of NAFLD in children and adolescents has increased remarkably, with the increasing trend of obesity [38]. A majority of NAFLD may develop into nonalcoholic steatosis hepatitis (NASH), promoting the pathogenesis of liver fibrosis and cirrhosis. Probiotic treatment is involved in the host–gut microbiota metabolic interactions implicated in the development of NAFLD [39].

Probiotics have been indicated to ameliorate NAFLD by regulating lipid metabolism and insulin resistance [40]. In the high-fat diet (HFD)-induced NAFLD mice, the oral administration of *Lactobacillus plantarum* ZJUIDS14 at the dose of 10^9^ U per day over 12 weeks significantly reduced hepatic steatosis and liver injury and reduced insulin resistance by activating the peroxisome proliferator-activated receptor alpha (PPARα)/AMP-activated protein kinase (AMPK) signaling pathway, and the composition of the gut microbiota, as well as the function of the intestinal barrier, was restored [18]. In addition, *Lactobacillus fermentum* CQPC06 alleviated NAFLD and improved the intestinal barrier function by stabilizing the gut–liver axis in mice. Moreover, it had a high resistance and survival rate in the gastrointestinal tract to exert its probiotic activity [19]. Similarly, the yogurt-derived *Lactobacillus plantarum* Q16 reduced the hepatic lipid content and improved the hepatic energy metabolism via the fibroblast growth factor 21 (FGF21)/adiponectin/AMPKα/peroxisome proliferator-activated receptor gamma coactivator 1-alpha (PGC-1α) signaling pathway [20]. In addition, *Lactobacillus rhamnosus* GG administration prevented NAFLD in rats by increasing their resistance to oxidative stress and inflammation [21]. Additionally, the treatment of *Lactobacillus lactis* and *Pediococcus pentosaceus* ameliorated NAFLD progression in mice by improving the main metabolic features, like short chain fatty acids (SCFAs), bile acids (BAs), and tryptophan metabolites, suggesting that the probiotics reprogrammed the gut microbiome and metabolic environment via the gut–liver axis [32]. VSL#3^®^ is a commercial product with a high concentration of probiotics containing one strain of *Streptococcus thermophilus* BT01, three strains of *Bifidobacteria*, and four strains of *Lactobacilli*. In a double-blind and placebo-controlled study, sixty patients with NAFLD were randomized to take VSL#3^®^ or a placebo for 3 months. The findings show that probiotic treatment significantly reduced the level of TG, high-sensitivity C-reactive protein (Hs-CRP), the hepatic aspartate transaminase (AST)/alanine transaminase (ALT) ratio, and the steatosis index (*p* < 0.05, baseline and placebo, respectively), and there were no significant differences in its effectiveness by gender [33]. The Mutaflor (R) probiotic with *Escherichia coli* Nissle 1917 was effective in alleviating the progression of NAFLD/NASH by regulating hepatic stellate cells and inhibiting Hedgehog and Hippo pathways in rats on high-fat high-sucrose diet (HFHSD) [28].

The intestinal metabolism of bile acids is essential for the gut–liver axis in NAFLD pathophysiology due to its interaction with the hepatic and intestinal farnesoid nuclear X receptor (FXR) [41,42]. A dysfunctional bile acid metabolism fails to activate the FXR and leads to increased lipogenesis, bile acid synthesis, and immune activation [43]. Hyodeoxycholic acid (HDCA), a type of gut microbiota-modified bile acid, effectively ameliorated NAFLD by inhibiting the FXR and increasing the level of hepatic *CYP7B1*, and it also improved the intestinal probiotics and promoted lipid catabolism via PPARα signaling [39]. Hepatic DNA methylation is also altered during the progression of NAFLD. The multi-omics machine learning approach connected the changes in the composition of gut microbiota, the metabolome, and liver DNA methylome in NAFLD patients. The findings demonstrate that the abundance of *Eubacterium siraeum* and potential probiotic *Blautia wexlerae* were increased in the gut microbiota profiles after fecal microbiota transplantation (FMT), and the hepatic DNA methylation profiles, especially threonyl-TRNA synthetase 1 (TARS) and zinc finger protein 57 (ZFP57), were altered. The probiotics isolated from FMTs might be developed into probiotic therapy for NAFLD via the modulation of hepatic DNA methylation [44].

In addition to the traditional probiotic products for the prevention of NAFLD, there are also some food ingredients, additives, and herbal compounds that exhibit probiotic activities. Food ingredients, like *Lactobacillus*-fermented black barley, food additives like gellan gum, and bioactive compounds like α-D-1,3-glucan from *Radix Puerariae thomsonii* and sinapine from rapeseed could ameliorate the progression of NAFLD through regulating gut microbiota and intestinal metabolites [45,46,47,48].

The potential benefits of probiotics on host health might not always be consistent and guaranteed. In a clinical study, a total of 72 NAFLD patients with over 30U/L of enzyme alanine aminotransferase (ALT) were randomly selected to receive probiotics containing six different *Lactobacillus* and *Bifidobacterium* species for six months. The consumption of probiotics displayed a more balanced gut microbiota structure and reduced inflammation in patients, but it failed to improve the small intestinal permeability significantly (circulating zonula occludens-1, probiotics −34.51 ng/mL ± 18.38 vs. placebo −33.34 ng/mL ± 16.62) [41]. In addition to the hepatic complications and severe liver injuries, NAFLD is a risk factor for cardiovascular diseases, which are the main causes of mortality [49,50]. The probiotic VSL#3^®^ was also investigated regarding its activity on the markers of cardiovascular risk and liver injury in patients with NAFLD. However, the intake of VSL#3^®^ could not improve the endothelial function and inflammation in patients with NAFLD [51]. Probiotic treatment has been widely used in the treatment of NAFLD; therefore, the viability and safety of new probiotic strains should be well-evaluated for the treatment and management of NAFLD in clinical studies to reduce uncertainty [52]. Additionally, further investigations could focus on the biochemical indexes of NAFLD-related diseases, like cardiovascular diseases, to reduce the risk of having other complications in patients with NAFLD.

## 3. Probiotics and Alcoholic Liver Disease

Excessive alcohol consumption is the main cause of alcoholic liver disease (ALD). Long exposure to alcohol can impair the intestinal barrier function and promote the production of endotoxins and lipopolysaccharides (LPSs) from the cell walls disrupted by Gram-negative bacteria. Both the dead bacteria and LPSs can be released from the cell walls and translocate to the liver, leading to alcoholic liver injury [53]. A large body of evidence has demonstrated that probiotic lactic acid bacteria treatment protects against ALD by reducing the alcohol-induced imbalances in gut microbiota, the level of LPSs reaching the liver, liver inflammation, and oxidative stress [54]. The intestine is known as the largest immune system, and the liver is also rich in innate immune cells, like natural killer (NK) cells; therefore, the importance of immune crosstalk to the function of the gut–liver axis is increasingly recognized [55,56]. Blood samples from a total of 103 patients with alcoholic hepatitis and cirrhosis were collected for flow cytometric analysis. The results indicate that the alternation of number, activation, and cytotoxicity of NK cells depended on the progression of ALD. The frequency of NKp46, an NK cell-activating receptor in NK cells, exhibited a negative association with the Maddrey discriminant function (MDF) score (r = −0.4285, *p* = 0.0073), a prognostic index for alcoholic hepatitis, and the frequency of NK cells were decreased in alcoholic cirrhosis. To confirm the effects of probiotics on ALD via the modulation of immune cells, EtOH-fed mice were treated with the probiotics *Phocaeicola dorei* and *Lactobacillus helveticus*; the probiotics ameliorated the ALD with a reduction in liver inflammation, improvement in the intestinal barrier integrality, and an increase in NK cell activity [34].

## 4. Probiotic and Nonalcoholic Fatty Steatohepatitis

Nonalcoholic fatty steatohepatitis (NASH) is characterized by severe hepatic inflammation and liver cell injury, which further has a risk of developing into cirrhosis, liver failure, and liver cancer [57,58]. Gut microbiota have been found to be an important factor in the pathogenesis of NASH, and targeting the gut–liver axis can be a promising therapeutic approach for NASH. *Lactobacillus plantarum* significantly improved the metabolic profiles in a choline-deficient HFD (CD-HFD)-induced NASH animal model. The top 10 gene set enrichment analysis (GSEA) pathways significantly enriched in *Lactobacillus plantarum*-treated mice showed 6 pathways related to the inflammation, indicating that the treatment led to the downregulation of inflammatory genes. Meanwhile, the metagenomic data and Kyoto Encyclopedia of Genes and Genomes (KEGG) database showed that the gut microbiome in the treated group mainly functioned in increasing L-arginine biosynthesis pathways, which contributed to the reduced inflammation by *Lactobacillus plantarum* in NASH [23]. In addition to *Lactobacilli*, other genera have also been investigated as the next-generation probiotics with therapeutic potential for NASH. According to the results of scRNA-seq in the liver tissues of patients, hepatic proinflammatory M1 macrophages (48.78% vs. M2 macrophages) and γδT cells (16.94% vs. other cells) were enriched in NASH patients. *Akkermansia muciniphila* supplementation reduced hepatic proinflammatory macrophages (M1) and toll-like receptor 2 (TLR2)-activated γδ T17 cells in HFD-induced NASH mice, which further modulated the macrophage polarization [29]. Moreover, the relative abundance of *Faecalibacterium prausnitzii* was found to decrease significantly in patients with NASH (*p* < 0.05). The supplementation *Faecalibacterium prausnitzii* attenuated the NASH symptoms by reducing the hepatic lipid content, alleviating the liver injuries and fibrosis, protecting against the gut barrier damage, and reducing the hepatic steatosis and liver inflammation [30]. Furthermore, treatment with the probiotic *Lactobacillus reuteri* and metronidazole, an antibiotic against *Bacteroidetes*, showed improvement in NASH via LPS/TLR4 and autophagy pathways, in which the regulation of *Bacteriodetes* and acetate might the main contribution [22]. Additionally, the mixture of probiotics, including nine species of *Lactobacilli,* four species of *Bifidobacteria*, and *Streptococcus salivarius* subsp. (*Thermophilus*), delayed the progression of NASH and attenuated fibrosis, as well as hepatic inflammation, in rats fed with an HSHF diet by regulating miR-1205 and the Hippo signaling pathway [35].

## 5. Probiotics and Hepatitis

Autoimmune hepatitis (AIH) is an inflammatory disease of the liver caused by an abnormal immune response to liver autoantigens, affecting people of any age [59]. The occurrence of AIH can be accompanied with gut microbiota dysbiosis, and it may further progress into cirrhosis and lead to liver failure. Probiotics have therapeutical potential to treat AIH by restoring the structure of gut microbiota [60]. *Bifidobacterium animalis* ssp. Lactis 420 was demonstrated to inhibit Th17 cell proliferation, reducing about 3% of Th17 cells in the liver and spleen, and it alleviated liver injury in mice with AIH by protecting the integrity of the intestinal barrier and inhibiting the receptor-interacting kinase 3 (RIP3) pathway [26]. In addition to the single-probiotic treatment, mixed probiotic compounds consisting of 15 strains from *Bifidobacterium*, *Lactobacillus*, and *Streptococcus* exhibited significant immunomodulatory activity by inhibiting hepatic inflammatory cell infiltration, reducing the percentage of Th1 and Th17 cells, and increasing the Treg cells in mice with AIH. In addition to improving the intestinal flora and gut barrier, the probiotics prevented the translocation of LPSs to the liver and inhibited the TLP4/kappa-light-chain-enhancer of the activated B cells (NF-κB) pathway [36]. The mixed probiotic compounds also attenuated concanavalin A (Con A)-induced AIH by regulating gut microbiota and maintaining the immune balance. However, the effects of synbiotics (probiotic and prebiotic, galactooligosaccharides, fructooligosaccharides, and inulin) were better than probiotics alone. Synbiotics had synergistic effects and enhanced the probiotic activity [61]. The prebiotic could increase the efficacy of delivering probiotics to the colon and reduce their degradation in the gastrointestinal tract, and it also helps maintain the microenvironment for probiotics to reproduce and act on the structure of gut microbiota [62]. Furthermore, koumine is the most abundant alkaloid in *Gelsemium elegans* Benth., a traditional Chinese medicine. It was found to ameliorate Con A-induced AIH by regulating the nuclear factor erythroid 2–related factor 2 (Nrf2) and NF-κB signaling pathways, and it also worked as a probiotic to increase the abundance of beneficial bacteria to improve liver health [63].

AIH is an immune-mediated disease, whereas viral hepatitis results from infections of specific hepatitis viruses (A, B, C, D, and E) [64,65]. The Hepatitis B virus (HBV) and HCV infection are the most common causes of acute and chronic liver diseases. HBV can present in patients’ blood and body fluids at a high concentration, and due to its stability at an ambient temperature, it can be transmitted via inapparent ways, like contaminated environmental surfaces, infected equipment, and exposure to nonintact skin [66]. The major transmission mode of HCV involves transfusion and/or parenteral contact with blood products. Perinatal transmission and contaminated needles, as well as sexual contact, are risk factors for HCV infection [67]. In addition to nonintact skin or infected blood products, HDV infection can occur via intrafamilial transmission and intravenous drug use [68]. Moreover, HEV is mainly transmitted via a fecal–oral route, such as contaminated water and raw infected meat, while parenteral and perinatal transmission are also implicated in HEV incidence [69]. The findings show that gut microbiota can be altered depending on the progression of viral hepatitis, and the abundance of some microbiota, including *Butyricimonas*, *Escherichia-Shigella*, *Lactobacillus*, and *Veillonella*, was significantly changed in patients with viral hepatitis, which could be considered markers for the risk of having viral hepatitis [70,71]. Treatment targeting gut microbiota in viral hepatitis, such as probiotics and FMT, has also achieved promising outcomes recently. Kefir, a traditional fermented milk made from kefir grains, mainly contains *Lactobacillus acidophilus*, *Bifidobacterium bifidum*, *Streptococcus thermophilus*, *Lactobacillus kefiranofaciens*, and *Leuconostoc* species [72]. An in vitro study showed that the treatment of Kefir at a dose of 800 µg/mL eradicated HBV and HCV thoroughly by decreasing the inflammation and regulating the immune activities, in which the metabolites were the main contributors to the antiviral activities of Kefir [73].

## 6. Probiotics and Cirrhosis

Cirrhosis is an advanced stage of liver disease and is accompanied with acute-on-chronic liver failure, which may induce acute decompensation, liver failure, and high mortality [74,75,76]. In clinical studies, probiotics have been proved to attenuate cirrhosis therapeutically by regulating gut microbiota. In a randomized controlled trial including 58 patients with compensated cirrhosis, the abundance of microbiome was increased, and the gut barrier function was improved after intaking multispecies probiotics (mainly containing *Bifidobacterium* and *Lactobacillus*) for 6 months. Patients receiving probiotics also exhibited increased innate immune response and gut permeability, in which the abundance of *Alistipes shahii* had a positive association with the level of neopterin (r_s_ = 0.354; *p* = 0.006), and the abundance of *Syntrophococcus sucromutans* and *Prevotella* sp. had a negative correlation with zonulin (r_s_ = −0.311, *p* = 0.018 and r_s_ = −0.285, *p* = 0.030, respectively) [37]. Additionally, a clinical study with 102 cirrhotic patients demonstrated that the oral intake of probiotics with a combination of *Bifidobacterium*, *Lactobacillus*, and *Enterococcus* improved the liver function in patients with cirrhosis, reduced the total bilirubin, and enhanced SCFA-producing bacteria [77]. Using metabolomic analysis, the probiotics mainly increased the level of glutamine (*p* = 0.002, FDR *p* = 0.007) and reduced the level of glutamate (*p* = 0.03, FDR *p* = 0.03), inducing an increase in the ratio of glutamine/glutamate (*p* = 0.009, FDR *p* = 0.01) in patients with cirrhosis, suggesting that the probiotic enhanced the ability of ammonia detoxification. Moreover, the high level of glutamine/glutamate was correlated with a lower abundance of *Paraprevotella* and *Oscillospira* [78]. It also benefited cognitive function and decreased inflammation in patients with cirrhosis [79].

On the other hand, clostridioides difficile infection (CDI) poses negative effects on many patients with cirrhosis. In a retrospective study, FMT was found successful in most CDI patients with cirrhosis, but some patients showed limited response to FMT treatment, and the utilization of probiotics might be one of the potential failure elements [80]. Some studies indicated that the use of probiotics may hinder the spontaneous recovery of the microbiome after antibiotic-induced perturbation [81,82].

## 7. Probiotics and Hepatocellular Carcinoma

Hepatocellular carcinoma (HCC) often occurs in the progressive stage of chronic liver diseases. The dysbiosis of gut microbiota may activate TLR4 and NF-κB signaling in the liver, promoting HCC initiation and progression. Mounting evidence from preclinical and clinical studies indicate that improvement in the gut microbiota structure is associated with reduced inflammation and the restoration of liver function, suggesting that the gut microbiota-targeted approach might result in better HCC outcomes [83]. Hence, probiotics might prevent HCC by contributing to a healthy condition of gut microbiota and the growth of beneficial bacteria [12]. It was found that the intake of probiotics reduced the incidence of HCC in patients with hepatitis B-related cirrhosis treated with antiviral medication [84,85]. *Streptococcus salivarius* probiotics isolated from human breast milk exhibited strong antioxidant activity and inhibitory activity on the proliferation of liver and breast cancer cells and are considered a potential source of probiotic functional food that protects against liver and breast cancers [86].

Increasing epidemiological evidence demonstrates that metabolic diseases, like NAFLD and type 2 diabetes (T2DM), have become the etiology underlying many cases of HCC [87]. *Bifidobacterium pseudolongum* was found to be the most depleted bacteria in the stools of mice with NAFLD-HCC, while the oral gavage of *Bifidobacterium pseudolongum* could significantly suppress the formation of NAFLD-HCC. Moreover, acetate was identified as the critical metabolite generated from *Bifidobacterium pseudolongum* in its conditioned media. It protected against NAFLD-HCC by secreting the antitumor metabolite acetate, which reached the liver via the portal vein and inhibited the interleukin 6 (IL-6)/Janus kinase 1 (JAK1)/signal transducer and activator of transcription 3 (STAT3) signaling pathway [27]. In addition, T2DM clinically increases the risk of developing HCC. The probiotic *Lactobacillus brevis* improved the health condition of T2DM mice and alleviated the disease T2DM-HCC progression by regulating the gut microbiota, in which *Actinomycetes* was considered the potentially important factor in the progression. Moreover, probiotic treatment downregulated the matrix metallopeptidase 9 (MMP9) and neurogenic locus notch homolog protein 1 (NOTCH 1) signaling pathways to prevent disease progression, indicating that probiotics could be used as adjuvant therapy for patients with T2DM and HCC [24].

In addition to being developed as therapy agents, probiotics are often used in the adjuvant treatment of immunotherapy for liver cancer to interfere with gut microbiota. However, the efficacy and side effects of immunotherapy for liver cancer could be affected by gut microbiota modulating the host immunity due to the different microbial profiles of recipients and the survival rate of probiotics through the gastrointestinal tract [88,89]. Therefore, modifications of gut microbiota should be evaluated and optimized to improve the treatment outcomes instead of over-usage.

## 8. Probiotics and Liver Transplantation

Liver disease is a global health issue, and the number of patients receiving liver surgery or transplantation goes up continuously [90]. Surgery may induce dysbiosis and lead to the limited outcome of surgical therapy. Patients have a risk of developing a bacterial infection after liver surgery, which may potentially result in liver failure and even mortality [91]. Numerous evidence illustrates that the perioperative use of probiotics contributes to lower infection rates and efficient prophylaxis against postoperative infections after liver surgery [91,92].

In liver transplantation, some immunosuppressive agents, like tacrolimus, are often used for the prevention of organ rejection post-transplant. The middle dosage (0.5 mg/kg) of tacrolimus treatment not only maintains the immunosuppressive activity and normal graft function but also the gut barrier integrity and improved the structure of gut microbiota with the increase in probiotics. It suggests that the dosage of immunosuppressive agents should be optimal for patients to exhibit normal graft function and probiotic activities [93]. However, some literature found that the health benefits of probiotics in liver transplantation are widely demonstrated in animal models, while the use of probiotics in clinical trials needs more investigation and consideration. A secondary analysis of a randomized trial showed that the pretransplant administration of 3 × 10^9^ colony-forming unit (CFU) probiotic capsules containing *Lactococcus lactis* PB411 (50.0%), *Lactobacillus casei* PB121 (25.0%), *Lactobacillus acidophilus* PB111 (12.5%), and *Bifidobacterium bifidum* PB211 (12.5%) had benefits on 6-month allograft function, including a decreased international normalized ratio (INR) for prothrombin time, c-reactive protein (CRP) concentration, and AST activity. However, the intake should not be longer than 30 days due to the potential negative effects with the increased levels of INR and γ-glutamyl transferase and abundance of *Bacteroides* and *Enterococcus* [92]. Therefore, probiotics may augment deleterious immune-mediated processes in patients. The further evaluation of the safety and efficacy of probiotic therapy is warranted, especially for those receiving liver transplantation surgery [94].

## 9. Probiotics and Cholestasis

Cholestasis is characterized by the abnormal production and transportation of bile, and gut microbiota are strongly related to the occurrence of cholestasis, in which bile acids play a pivotal role [8]. Cholestasis can be classified into two main types, intrahepatic and extrahepatic cholestasis. Primary sclerosing cholangitis (PSC), a chronic and progressive cholestatic liver disease, is mainly characterized by intrahepatic or extrahepatic strictures and biliary fibrosis [95]. *Pediococcus pentosaceus* Li05 is a probiotic with anti-inflammatory activity, and it was used to treat 3,5-diethoxycarbonyl-1,4-dihydrocollidine (DDC)-induced PSC in mice. The findings show that the intake of Li05 alleviated liver damage, hepatic inflammation, fibrosis, and bile duct hyperplasia. The treatment inhibited hepatic bile acid synthesis and transport by activating FXR- small heterodimer partner (SHP) and the ileal FXR-FGF15 signaling pathway. The intestinal barrier was also improved to reduce the level of bacterial endotoxin [31]. Additionally, intrahepatic cholestasis in pregnancy (ICP) is a reversible form of cholestasis in late pregnancy, and it is characterized by increased bile acid levels in maternal serum [96]. A cholestatic pregnancy might result in the enhanced susceptibility of the offspring to inflammation via bile acid metabolism and gut microbiota. The probiotic *Lactobacillus rhamnosus* LRX01 suppressed FXR expression in the ileum and improved the immune function of offspring, reducing susceptibility to LPS exposure [25]. Furthermore, some drugs can induce cholestasis-related liver injury [97]. *Lactobacillus casei* had previously been reported to alleviate negative gastrointestinal symptoms, such as vomiting and appetite loss, during tuberculosis treatment, which was usually associated with tuberculosis-drug-induced liver injury [98]. In addition, the increased levels of alkaline phosphatase (ALP) and bilirubin are characteristics of cholestasis-type liver injury. In a clinical study, *Lactobacillus casei* supplementation significantly reduced the level of ALP and bilirubin (0% vs. 4.9%, *p* = 0.024; 1.2% vs. 9.7%, *p* = 0.013, respectively) compared to untreated patients receiving tuberculosis-drug therapy. Moreover, it attenuated the abnormal cholestasis-related liver indices by reducing the serum level of LPSs, improving the intestinal barrier function, and regulating the gut microbiota, in which the abundance of *Bacteroidetes* was decreased and that of *Actinobacteria* and *Firmicutes* was elevated [99]. Although most evidence little reported the toxicity of probiotic treatments, their usage should be considered in certain populations, like pregnant patients, neonates born prematurely, and individuals with immune deficiency.

## 10. Conclusions

Gut microbiota is a crucial factor in the pathogenesis of multiple chronic diseases, and gut microbiota therapy, like probiotics, still exhibits high scientific and commercial value. Research over the past few years has demonstrated the efficacy of probiotic treatment on hepatobiliary diseases, such as NAFLD, ALD, NASH, AIH, cirrhosis, and HCC, liver transplantation, and cholestasis. In addition to its therapeutical potential, more updated research has focused on the safety, viability, and stability of different strains of probiotics. It is also essential to optimize the dosage and medication duration. Future research could focus on the specific mechanism of probiotics and their limitations in clinical trials and explore more widely applicable genera or strains of probiotics in the management and treatment of hepatobiliary diseases.

## Figures and Tables

**Table 1:** Preclinical and clinical studies on different strains of probiotics for treating liver diseases.

Strains	Type of Diseases	Subjects	Dosage	Duration	Effects	Modifications of Gut Microbiota	Molecular Mechanism	Ref.
*Lactobacillus plantarum* ZJUIDS14	NAFLD	Mice	10^9^ CFU/day	12 weeks	Mitigated hepatic steatosis and liver damage induced by HFD. Reduced insulin resistance. Improved mitochondrial function.	↑ *Coprostanoligenes* group, *Ruminococcaceae UCG-014*, *Allobaculum*, *and Ruminiclostridium 1*. ↓ *Roseburia*.	↑ PPARα, AMPK. ↑ DRP1, UQCRC2, MTCO1, SDHB, and NDUFB8.	[18]
*Lactobacillus fermentum* CQPC06	NAFLD	Mice	10^9^ CFU/kg	8 weeks	Increased intestinal barrier strength. Reduced liver inflammation. Regulated liver lipid metabolism disorder. Reduced insulin resistance.	↑ *Bacteroides* and *Akkermansiain*. ↓ The ratio of *Firmicutes*/*Bacteroides*.	↑ PPARα, CYP7A1, CPT1, and LPL. ↓ PPARγ and C/EBPα. ↑ ZO-1, occludin, and claudin-1. ↓ CD36.	[19]
*Lactobacillus plantarum* Q16	NAFLD	Mice	10^9^ CFU/mL	8 weeks	Decreased serum and hepatic lipid profile. Improved hepatic energy metabolism.	↓ *Proteobacteria* and the ratio of *Firmicutes*/*Bacteroidetes*. ↑ S24-7 and *Lactobacillaceae*. ↓ *Rikenellaceae*, *Lachnospiraceae*, *Odoribacteraceae*, and *Desulfovibrionaceae*.	↓ FAS, ACC, SCD-1, SREBP-1c, and ATGL. ↑ CPT1α and PPARα.	[20]
*Lactobacillus rhamnosus* GG	NAFLD	Rats	10^9^ CFU/day	8 weeks	Decreased the level of hepatic triglyceride. Reduced liver oxidative stress and inflammation.	/	/	[21]
*Lactobacillus reuteri* (in combination with metronidazole)	NASH	Rats	2 × 10^9^ CFU/day	8 weeks	Declined the number of TLR4- and LC3Ⅱ-positive staining hepatocytes. Benefited the hepatic lipid profile. Alleviated liver inflammation and autophagy. Regulated acetate/propionate/butyrate ratios.	↓ The ratio of *Firmicutes*/*Bacteroides*.	↓ LPS, NF-κB, and TNF-α. ↓ mTOR and p-Akt.	[22]
*Lactobacillus plantarum*	NASH	Mice	10^9^ CFU/day	12 weeks	Attenuated liver inflammation. Improved insulin tolerance and hepatic lipid content. Upregulated L-arginine synthesis.	/	/	[23]
*Lactobacillus brevis*	T2DM-HCC	Mice	10^8^, 10^9^, 10^10^ CFU/mL	201 days	Improved glucose homeostasis. Relieved insulin resistance and pancreatic and liver damage. Reduced inflammation.	↓ *Actinomycetes*.	↓ MMP9, NOTCH 1, and Hes1.	[24]
*Lactobacillus rhamnosus* LRX01	ICP	Rats	2 × 10^8^ CFU/day	7 days	Declined susceptibility to LPS exposure in ICP offspring.	/	↓ FXR. ↓ RORγ-T, Foxp3, and NLRP3.	[25]
*Bifidobacterium animalis* ssp. Lactis 420	AIH	Mice	10^9^ CFU/day	4 weeks	Increased fecal SCFA content. Improved intestinal barrier function. Declined the level of endotoxin in the serum. Decreased the percentage of Th17 cells in liver and spleen.	↓ *Bacteroides* and *Ruminococcus*. ↑ *Lactobacillus*.	↑ Occludin and ZO-1. ↓ TNF-α, IL6, and IL-1β. ↓ RIP and MLKL. ↓ CCL2 and CCR2.	[26]
*Bifidobacterium pseudolongum*	NAFLD-HCC	Mice	1 × 10^8^ CFU/day	26/28 weeks	Protected against NAFLD-HCC progression formation. Improved the gut barrier function. Inhibited the proliferation of NAFLD-HCC cells. Increased the level of acetate.	↑ *Bifidobacterium choerinum*, *Bifidobacterium animalis*, and *Oscillibacter* sp. PEA192. ↓ *Paeniclostridium sordellii* and *Enterobacter hormaechei*.	↑ GPR43. ↓ IL-6, p-JAK1, and p-STAT3.	[27]
*Escherichia coli* Nissle 1917	NAFLD	Rats	10^9^ CFU/mL	12 weeks	Normalized biochemical and histopathologic profiles caused by NAFLD/NASH. Suppressed Hedgehog and Hippo pathways.	/	↑ *RPARP-AS-1* lncRNA. ↓ *miR-650*, *FOXA2*, and *TEAD2* mRNA. ↑ *LATS2* mRNA.	[28]
*Akkermansia muciniphila*	NASH	Mice	10^9^ CFU/day	20 weeks	Reduced the inflammation. Modulated the macrophage polarization. Protected against intestinal barrier failure.	/	↓ TNF, TLR1, and TLR2.	[29]
*Faecalibacterium prausnitzii*	NASH	Mice	10^8^ CFU/day	8 weeks	Alleviated the hepatic lipid content, liver injuries and fibrosis, gut barrier damage, hepatic steatosis, and liver inflammation.	/	↓ CD36, FATP5, PPAR-𝛾, SREBP-1c, FAS, and LPL.	[30]
*Pediococcus pentosaceu* Li05	PSC	Mice	2 × 10^8^ CFU/day	14 days	Attenuated liver damage and inflammation. Relieved liver fibrosis and bile duct hyperplasia. Inhibited bile acid synthesis. Enhanced the secretion of fecal bile acid. Increased the concentration of SCFAs and gut barrier integrity.	↑ *Pediococcus*, *Anaerostipes*, and *Eubacterium siraeum*.	↑ FGF15, and FXR. ↓ *ABST*, *OSTα*, *OSTβ*, and *MRP2* mRNA.	[31]
*Lactobacillus lactis* and *Pediococcus pentosaceus*	NAFLD	Mice	10^9^ CFU/day	8 weeks	Normalized weight ratio and NAFLD biochemical profile. Restored gut tight junction. Regulated tryptophan pathway.	↓ The ratio of *Firmicutes*/*Bacteroides*. ↓ *Helicobacter*, *KE159600_g*, *Mucispirillum*, *Pseudoflavonifractor*, *Clostridium_g21*, and *Faecalibaculum*. ↑ *Lactobacillus*.	↓ MAPK signaling pathway.	[32]
*Streptococcus thermophilus* BT01, *Bifidobacteria*, and *Lactobacilli*	NAFLD	60 patients	2 sachets/day	3 months	Ameliorated hepatic parameters and echography grading.	/	/	[33]
*Phocaeicola dorei* and *Lactobacillus helveticus*	ALD	Mice	10^9^ CFU/day	10 weeks (three times a week)	Reduced liver inflammation and intestinal barrier damage. Increased NK cell activity.	/	↓ p-MAPK, p-38, and p-ERK. ↑ Occludin, claudin, and ZO-1.	[34]
*Lactobacilli, Bifidobacteria* and *Streptococcus salivarius* subsp. *(Thermophilus)*	NASH	Rats	4 × 10^9^ CFU/kg b.w./day	12 weeks	Ameliorated steatosis, inflammation, and fibrosis grades.	/	↓ *YAP1* mRNA and *miR-1205*. ↑ *LATS1* and *NF2* mRNAs. ↑ *SRD5A3-AS1* lncRNA. ↓ IL-6 and TGFβ-1. ↓ α-SMA. ↑ LATS1/2.	[35]
15 strains from *Lactobacillus, Bifidobacterium,* and *Streptococcus*	AIH	Mice	10^9^ CFU/day	42 days	Alleviated liver injury. Inhibited hepatic inflammatory cell infiltration. Maintained intestinal barrier integrity. Suppressed the translocation of LPS. Increased SCFA concentration.	↓ The ratio of *Firmicutes*/*Bacteroides*. ↑ *Verrucomicrobia* and *Actinobacteria*. ↓ *Saccharibacteria*, *Deferribacteres*, and *Proteobacteria*.	↓ IL-17A and IFN-γ. ↑ TGF-β. ↑ Occludin and ZO-1. ↓ TLR4, NF-κB, p-IκB/IκB, Bax, and caspase-3.	[36]
9 strains from *Lactobacillus* and *Bifidobacterium*	Cirrhosis	58 patients with compensated cirrhosis	1.5 × 10^10^ CFU/day	6 months	Increased the level of neopterin. Decreased the fecal levels of zonulin.	↑ *Faecalibacterium prausnitzii*, *Syntrophococcus sucromutans*, *Bacteroides vulgatus*, *Alistipes shahii*, and *Prevotella* species.	/	[37]

ACC, acetyl-CoA carboxylase; AMPK, AMP-activated protein kinase; ASBT, apical sodium-dependent bile acid transporter; α-SMA, alpha smooth muscle actin; ATGL, adipose triglyceride lipase; Bax, Bcl2-associated X protein; CCL2, C-C motif chemokine ligand 2; CCR2, C-C motif chemokine receptor 2; CD36, CD36 molecule; C/EBPα, CCAAT enhancer-binding protein alpha; CPT1, carnitine palmitoyltransferase I; CYP7A1, cytochrome P450 family 7 subfamily A member 1; DRP1, dynamin-related protein 1; FAS, fatty acid synthase; FATP5, fatty acid transport protein-5; FGF15, fibroblast growth factor 15; FOXA2, forkhead box A2; Foxp3, forkhead box P3; FXR, farnesoid X receptor; GPR43, G protein-coupled receptor 43; Hes1, hairy and enhancer of split-1; IFN-γ, interferon gamma; IκB, inhibitor of nuclear factor kappa B; IL-6, interleukin 6; LATS2, large tumor suppressor kinase 2; LncRNAs, long noncoding RNAs; LPL, lipoprotein lipase; MAPK, mitogen-activated protein kinase; miR-650, microRNA-650; MMP9, matrix metallopeptidase 9; MRP2, multidrug resistance-associated protein 2; MTCO1, mitochondrially encoded cytochrome c oxidase I; mTOR, mammalian target of rapamycin; MLKL, mixed lineage kinase domain like pseudokinase; NDUFB8, NADH/ubiquinone oxidoreductase subunit B8; NF2, neurofibromatosis type 2; NLRP3, NLR family pyrin domain containing 3; NOTCH 1, neurogenic locus notch homolog protein 1; OSTα, organic solute transporter subunit alpha; p-Akt, phospho Akt kinase; p-ERK, phospho-extracellular signal-regulated kinase; p-JAK1, phospho-Janus kinase; PPARα, peroxisome proliferator-activated receptor alpha; p-STAT3, phospho-signal transducer and activator of transcription 3; RIP, receptor-interacting protein; RORγ-T, retinoic acid receptor-related orphan receptor gamma; SCD-1, stearoyl-coenzyme A desaturase 1; SDHB, succinate dehydrogenase complex iron sulfur subunit B; SRD5A3-AS1, steroid 5 alpha-reductase 3-antisense RNA 1; SREBP-1c, sterol regulatory element-binding protein 1; TEAD2, TEA domain transcription factor 2; TGF-β1, transforming growth factor beta 1; TLR1, toll-like receptor 1; TNF-α, tumor necrosis factor-α; UQCRC2, ubiquinol-cytochrome c reductase core protein 2; YAP1, Yes1-associated transcriptional regulator; ZO-1, zonula occludens-1.
